# Botanical drugs and their natural compounds: a neglected treasury for inhibiting the carcinogenesis of pancreatic ductal adenocarcinoma

**DOI:** 10.1080/13880209.2024.2421759

**Published:** 2024-11-09

**Authors:** Yunfei Dai, Xi Guan, Fangyue Guo, Xin Kong, Shuqi Ji, Dong Shang, Changchuan Bai, Qingkai Zhang, Liang Zhao

**Affiliations:** a Department of General Surgery, The First Affiliated Hospital of Dalian Medical University, Dalian, China; b Institute (College) of Integrative Medicine, Dalian Medical University, Dalian, China; c Laboratory of Integrative Medicine, The First Affiliated Hospital of Dalian Medical University, Dalian, China; d College of pharmacy, Dalian Medical University, Dalian, China

**Keywords:** Pancreatic ductal adenocarcinoma, inflammatory microenvironments, pancreatitis, botanical drugs, natural compounds

## Abstract

**Context:**

Pancreatic ductal adenocarcinoma (PDAC), which is characterized by its malignant nature, presents challenges for early detection and is associated with a poor prognosis. Any strategy that can interfere with the beginning or earlier stage of PDAC greatly delays disease progression. In response to this intractable problem, the exploration of new drugs is critical to reduce the incidence of PDAC.

**Objective:**

In this study, we summarize the mechanisms of pancreatitis-induced PDAC and traditional Chinese medicine (TCM) theory and review the roles and mechanisms of botanical drugs and their natural compounds that can inhibit the process of pancreatitis-induced PDAC.

**Methods:**

With the keywords ‘chronic pancreatitis’, ‘TCM’, ‘Chinese medicinal formulae’, ‘natural compounds’, ‘PDAC’ and ‘pancreatic cancer’, we conducted an extensive literature search of the PubMed, Web of Science, and other databases to identify studies that effectively prevent PDAC in complex inflammatory microenvironments.

**Results:**

We summarized the mechanism of pancreatitis-induced PDAC. Persistent inflammatory microenvironments cause multiple changes in the pancreas itself, including tissue damage, abnormal cell differentiation, and even gene mutation. According to TCM, pancreatitis-induced PDAC is the process of ‘dampness-heat obstructing the spleen and deficiency due to stagnation’ induced by a variety of pathological factors. A variety of botanical drugs and their natural compounds, such as Chaihu classical formulae, flavonoids, phenolics, terpenoids, etc., may be potential drugs to interfere with the development of PDAC *via* reshaping the inflammatory microenvironment by improving tissue injury and pancreatic fibrosis.

**Conclusions:**

Botanical drugs and their natural compounds show great potential for preventing PDAC in complex inflammatory microenvironments.

## Introduction

Pancreatic cancer (PC), often called the ‘king of cancers’, is in fact mostly pancreatic ductal adenocarcinoma (PDAC), accounting for approximately 90% of total cases, with a mere 12% five-year relative survival rate (Ordóñez 2001; Zhang W. et al. 2023; Wang et al. 2021; Siegel et al. 2023). Risk factors for PDAC include individual characteristics [age (older), gene mutations and race (black), etc.], lifestyle and environment [exposure to certain heavy metals, excessive alcohol consumption, smoking and obesity, etc.], and disease status [pancreatitis and diabetes mellitus, etc.] (Cai et al. 2021; Yadav and Lowenfels 2013). Patients with chronic pancreatitis (CP) or recurrent acute pancreatitis (AP) are substantially more likely to develop PC, according to mounting evidence (Kirkegård et al. 2017). Tissue cells exposed to chronic inflammation undergo changes and interactions at various levels, such as metabolism, inflammatory cytokines, and immunity (Zhao et al. 2021; Padoan et al. 2019). Questions remain regarding the mechanisms involved in stimulation of different types of pancreatic cells, repair of pancreatic tissue, and characteristics of precancerous lesions in the presence of CP; thus, the current clinical strategies for treating PDAC are insufficient (Strobel et al. 2019; Vreeland and Katz 2018; Beutel and Halbrook 2023). On the one hand, the inherent concealment of PDAC poses a great challenge to early surgical intervention; on the other hand, pancreatic cancer cells (PCCs) not only develop internal drug resistance but also develop a thick fibrous matrix around them, and other factors hinder the effectiveness of the first-line chemotherapy drug gemcitabine. Currently, exploring new drugs to overcome existing shortcomings is a difficult challenge. Within this dire situation, basic and clinical research on pancreatic inflammation and cancer is progressively influenced by the use of botanical drugs and their natural compounds, including but not limited to traditional Chinese medicine (TCM). However, from the perspective of modern medicine, the application of TCM is still mysterious because of its rich sources and complex components, as well as the ‘quality-dose-efficacy’ relationship in the complex system of TCM. In fact, actively exploring the function of TCM preparations may benefit the development of new therapies based on integrated medicine. In this review, we first provide an understanding of the effects of TCM on pancreatitis-induced PDAC according to the possible pathological mechanisms involved in this process. Moreover, the experimental results concerning the potential blocking effects of botanical drugs and their natural compounds on the malignant transformation of pancreatitis, including relevant models, therapeutic effects, and mechanisms, are detailed.

## TCM understanding of pancreatitis-induced PDAC

There is no record of the pancreas as an organ in TCM. Based on its anatomical position, morphology, physiological function, etc., we classify the pancreas as the spleen in TCM, that is, ‘the pancreas and spleen as a whole’. In detail, the concept of the ‘spleen’ in TCM not only refers to the spleen itself in modern medicine but also includes the pancreas. As an organ with dual endocrine and exocrine capabilities (Karpińska and Czauderna 2022), the pancreatic islets in the endocrine tissue mainly secrete insulin and glucagon to participate in regulating the metabolism of the 3 major nutrients, sugar, fat and protein, which corresponds to the ‘transportation’ of the ‘spleen’ in TCM; that is, the transportation and distribution of qi, blood, essence, fluid and other nutrient substances to the whole body. The pancreatic enzymes secreted by the exocrine tissue of the pancreas are released into the duodenum through the pancreatic duct and directly participate in the digestion of food, which corresponds to the ‘transformation’ of the ‘spleen’ in TCM, assisting the stomach and intestines in decomposing, digesting and absorbing nutrients.

The occurrence of PDAC is the consequence of the combination of genetic mutations and the inflammatory microenvironment (Clarke 2019; Hu et al. 2023; Alonso-Curbelo et al. 2021). Pancreatitis initiates oxidative stress (OS) (Nagao et al. 2016; Ren et al. 2020; Schoenberg et al. 1995), which is characterized by excessive production of reactive oxygen species (ROS) and reactive nitrogen species, disrupting the balance between oxidation and antioxidation (Sies et al. 2017; Pizzino et al. 2017). Excessive ROS and other free radicals cause DNA damage in both the nucleus and mitochondria, resulting in an increase in oxidative DNA levels and chromosomal translocation. One significant stage in the emergence of cancer is the mutation of genes (Cadet and Wagner 2013; Valko et al. 2006; Cheng et al. 2023; Ragu et al. 2007). Moreover, pancreatitis induces dysfunctions in pancreatic endocrine and exocrine secretion and reduces the physiological functions of ‘transportation’ and ‘transformation’ of the ‘spleen’ in TCM, leading to pathological manifestations such as steatosis, intensified inflammation, OS, and impaired pancreatic microcirculation (Jaworek et al. 2005). Persistent inflammation forces adaptive changes in acinar cells, ductal cells, pancreatic stellate cells (PSCs), and inflammatory leukocytes within the pancreas to form a protumour inflammatory microenvironment (Murtaugh and Keefe 2015). PSCs transition into an activated state because of their location downstream of inflammatory or damaged cells, which respond to various signalling factors in a continuously damaged tissue environment (Sherman 2018). As the leading source of the extracellular matrix (ECM), activated PSCs generate a large amount of α-smooth muscle actin (α-SMA), collagen (Col), fibronectin (Fn), etc. (Wang, Chen, et al. 2024), leading to pancreatic fibrosis (PF), a unique histopathological feature of pancreatitis. The activation mechanism of PSCs involves various pathological reactions, such as pancreatic duct hypertension, that can be induced by pancreatitis, (Swain et al. 2022), ‘fibrotic factors’ (Laklai et al. 2016; Ng et al. 2022), disordered lipoprotein metabolism (Yang et al. 2022), and pancreatic peripheral circadian rhythm disorders (Jiang et al. 2022). In addition, PSCs can be activated and differentiated into cancer-associated fibroblasts (CAFs). Myofibroblasts, a subtype of CAFs, are found close to the edge of the tumour and express matrix metalloproteinases (MMPs), tissue inhibitors of metalloproteinases (TIMPs), and α-AMS. They are also more capable of proliferating and migrating. Invasive CAFs can also secrete inflammatory cytokines such as interleukin-6 (IL-6) away from tumours (Biffi et al. 2019; Öhlund et al. 2017; Masamune et al. 2009; Li et al. 2011).

**Figure 1.**
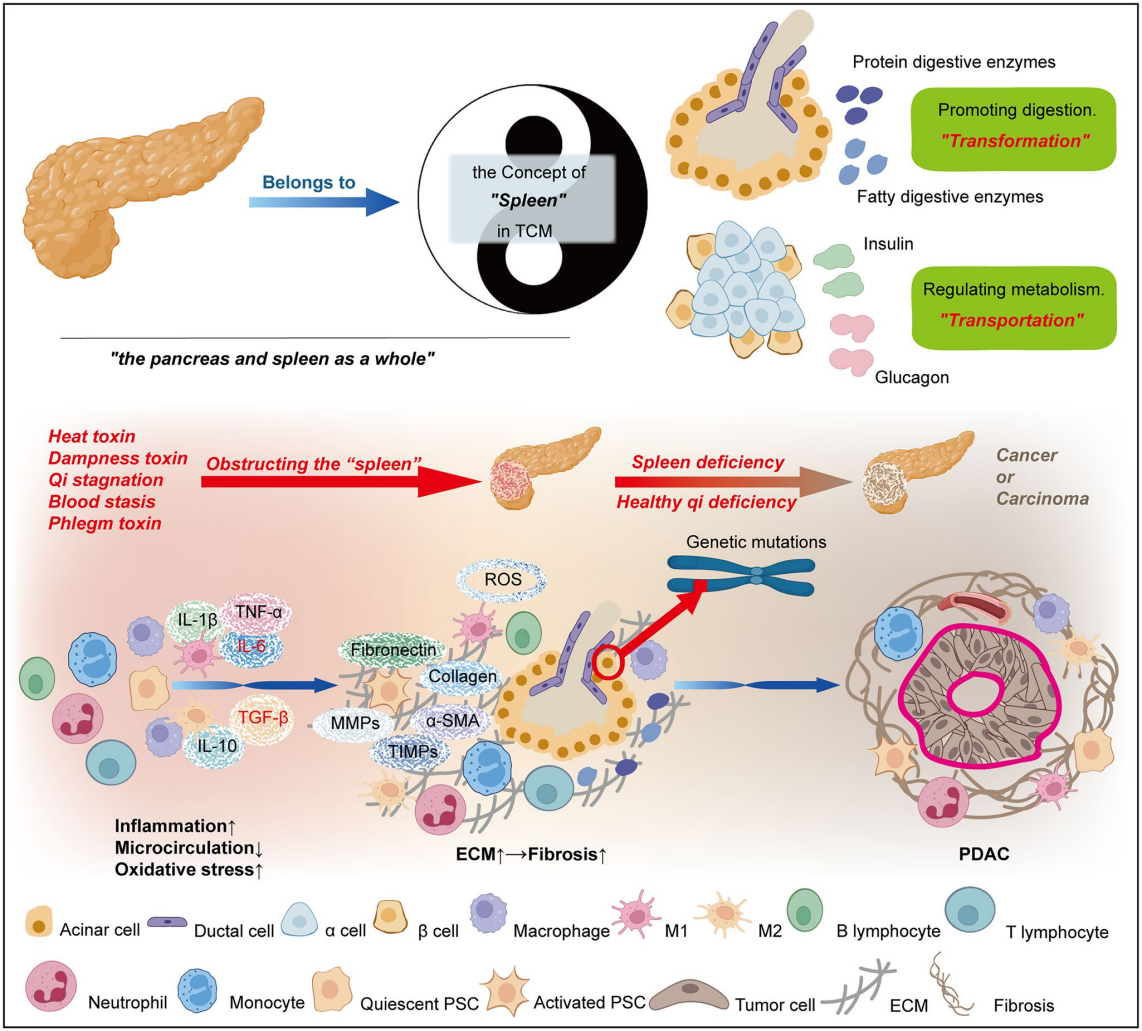
Based on the viewpoint of ‘the pancreas and spleen as a whole’ in TCM, pancreatitis-induced PDAC can be interpreted from the perspectives of ‘Dampness-heat obstructing the spleen and deficiency due to stagnation’.

Pancreatitis-induced PDAC can be summarized as the forced response of the transgenic pancreas to the inflammatory microenvironment. In the state of long-term inflammation and fibrosis leading to pancreatic damage (de Rijk et al. 2023; Cañamares-Orbís et al. 2022), patients often exhibit poor nutritional status, including emaciation, weakness, and weakened immunity. This is due to qi stagnation, heat toxin, dampness toxin, and phlegm toxin retention, resulting in the weakness of healthy qi. The inflammatory and cancerous microenvironments have direct effects on the pancreas in addition to the growth, proliferation, and migration of the tumour cells themselves. Various components play a role, such as PSCs, fibroblasts, macrophages, T lymphocytes, vascular endothelial cells, adipocytes, and other cell types, as well as ILs and transforming growth factor-β (TGF-β), vascular endothelial growth factors (VEGFs), adipokines and other factors (Hao et al. 2017; Quail and Joyce 2013; Balkwill et al. 2012). Overall, the general view of the TCM theory on the transformation of the pancreas from inflammation to cancer is the deficiency of healthy qi and the accumulation of pathogenic qi. Specifically, dampness-heat obstructs the spleen and leads to deficiency due to stagnation, qi movement disorders and hyperactive cancer toxins. This is a key pathological mechanism recognized by TCM for the transformation of the pancreas from inflammation to cancer (Figure 1).

When TCM is employed for the management of pancreatitis, the formulation of treatment strategies is typically customized according to the aetiology and pathogenesis characteristics, the patient’s clinical symptoms and specific areas of pain. To prevent the progression of pancreatitis-induced PDAC, TCM posits a multitude of potential therapeutic interventions that correspond to the etiopathogenic factors depicted in Figure 1. Herbs characterized by a cold nature and bitter taste are strategically deployed to clear heat and dry dampness, effectively targeting the underlying pathomechanism of ‘dampness-heat encumbering the spleen and the deficiency stemming from stagnation’. Additionally, the judicious use of herbs that facilitate blood circulation and dissolve blood stasis is pivotal for ameliorating local microcirculatory dynamics. This nuanced approach is of considerable significance for the precise identification of the core pathomechanisms involved in pancreatitis-induced PDAC and for the rational design of targeted pharmaceutical prescriptions.

## Natural compounds of botanical drugs for inhibiting the precursors of PDAC: ADM and/or PanIN

Acinar-to-ductal metaplasia (ADM) and pancreatic intraepithelial neoplasia (PanIN) lesions, as precursors of PDAC, are highly important for inhibiting the occurrence and development of PDAC. The inhibitory effects of natural botanical drugs on ADM and PanIN lesions are detailed in Table 1.

**Table 1.** Potential effects of the metabolites of the natural compounds of botanical drugs for inhibiting the precursors of PDAC: ADM and/or PanIN.

Compound category	Compounds	Study design	Dosage of administration	Efficacy	Possible mechanism	Refs
Flavonoids	Quercetin	C57BL/6 mice injected caerulein for CP	65 mg/kg/day for 8 weeks	↓Tissue damage; ↓ADM and PanIN lesions; ↓Inflammation; ↓Activation of PSCs;	↓5-HT, RhoA, ROCK1, ROCK2, α-SMA, NF-κB (in nuclear)	(Tao et al. 2020)
		Primary acinar cells treated with 5-HT	50 μΜ for 3 days and 5 days	↓ADM formation	↓CK19	(Tao et al. 2020)
	Baicalein	Murine macrophages cells RAW264.7 treated with LPS	10 μM, 20 μM, 40 μM for 18 h	Improved inflammatory microenvironment	↓TNF-α, NO	(Pu et al. 2018)
		Rat exocrine pancreas cells AR42J treated with macrophage-conditional medium or recombinant rat TNFα	40 μM for 5 days	↓ADM lesions	↓CK-19, TNF-α, degradation of IκBα, p-IκBα, p65 translocation to the nucleus; ↑Amylase/CK-19;	(Pu et al. 2018)
Phenolics	Resveratrol	*LSL-Kras^G12D/+^; Pdx1-Cre* (KC) mice injected with cerulein	50 mg/kg/day for 6 months	↓Tissue damage; ↓ADM and PanIN lesions; ↓PC progression from low-grade lesions to high-grade lesions; ↓Inflammation; ↓PF; ↓Col deposition;	↓Col, the number of Ki67^+^ proliferative cells, p-IKK, p-IKB, p-NF-κB, MMP9, p-ERK (cytoplasm), p-STAT3 (nuclear), Cox2, Bcl2	(Qian et al. 2020)
		Pancreatic acinar cells cultured in 3D conditions treated with TNF-α	50 μM for 5 days	↓Proliferation of pancreatic acinar cells/precancerous cells; ↓Formation of duct-like structures;	↓CyclinD1, MMP9, Cox2, Bcl2, CK19, p-NF-κB; ↑amylase;	(Qian et al. 2020)
Alkaloids	Capsaicin	*LSL-Kras^G12D^/Pdx1-Cre* C57BL/6J mice injected caerulein for CP	10 p.p.m. and 20 p.p.m. in diet fed for 8 weeks	↓Tissue damage; ↓ADM and mPanIN lesion formation and progression; ↓Progression of PanIN-1 to high-grade PanIN-2 and PanIN-3; ↓Inflammatory cell infiltration; ↓PF;	↓Pancreas weight, ratio of pancreas/BW, the number of MPO- and Mac-3-positive cells, PCNA-labeled (proliferating cell nuclear antigen) cells, p-ERK, p-c-Jun, Shh, Gli 1	(Bai et al. 2011)
	Berberine	Male C57BL/6 mice injected caerulein for CP	200 mg/kg once per day, 3 days per week for 8 weeks (pretreatment) and 4 weeks (treatment)	↓ADM and PanIN lesions and development; ↓Glycolysis; ↓PF;	↓α-SMA, CK19, lactate, glucose consumption, LDHA, ALDOA, PFKL, PKM2, PDK1, p-mTOR, HIF-1α; ↑Amylase, lipase, p-AMPK;	(Liu et al. 2022)
		Pancreatic acinar cells treated with TGF-β for acinar-to-ductal cell differentiation	10 μM for 1 day	↓PanIN development	↓CK19; ↑Amylase;	(Liu et al. 2022)

Note: ↓: Decrease or downregulate; ↑: Increase or upregulate.

## Quercetin

Plants are an excellent source of quercetin (C_15_H_10_O_7_), which is primarily found in *Allium cepa L.* (onions) and *Malus domestica (*Suckow) Borkh. (apples), and *Camellia sinensis (*L.) Kuntze (tea). It is intimately linked to the prevention and treatment of cancers owing to its strong antioxidant and anti-inflammatory properties(Tang et al. 2020). Research has shown that quercetin can effectively reduce the expression of the ductal marker cytokeratin 19 (CK19) and inhibit 5-hydroxytryptamine (5-HT)-induced ADM and PanIN lesions. Moreover, it can effectively inhibit the activation of PSCs (Tao et al. 2020).

## Baicalein

*Scutellaria baicalensis* Georgi (Huangqin), which is rich in the bioactive flavone baicalein (C_15_H_10_O_5_), is a paradigmatic medicinal agent because of its heat-clearing and dampness-drying properties. Baicalein has anticancer activity and extremely low toxicity to normal cells. It can inhibit the activity of various tumour cells and regulate immune cells, endothelial cells, fibroblasts, and other tumour stromal cells and the ECM; thus, it is essential for the development, spread, and metastasis of tumours (Wang, Wang et al. 2024). Baicalein can improve the inflammatory microenvironment and inhibit the activation of nuclear factor kappa-B (NF-κB) in pancreatic acinar cells and may also have an indirect inhibitory effect on the occurrence of ADM lesions through the suppression of the proinflammatory activity of macrophages (Pu et al. 2018). However, due to the poor hydrophilicity, low solubility, and poor oral bioavailability of baicalein, its clinical application is greatly limited (Wang, Wang et al. 2024).

## Resveratrol

Resveratrol (C_14_H_12_O_3_) is known as a plant antitoxin, mainly in plants such as *Polygonum cuspidatum* Sieb. et Zucc. (Huzhang) and *Vitis vinifera* L. (grape). It has been proven to have varying degrees of therapeutic effects on diseases such as type 2 diabetes, obesity and various cancers by regulating a variety of cell signalling molecules (Singh et al. 2019). In the initial phases of the disease, resveratrol reduces the formation of ADM and PanIN, whereas as the disease progresses, its ability to slow down the progression of low-grade lesions to high-grade lesions and reduce the occurrence of CP-mediated PDAC through suppression of the NF-κB signalling pathway in the precancerous lesions of patients with PDAC increases (Qian et al. 2020).

## Capsaicin

*Capsicum frutescens* L. is widely cultivated and consumed worldwide because of its spicy taste and is often used for eating vegetables, condiments, etc. Capsaicin (C_18_H_27_NO_3_) is one of the chemicals that endows *Capsicum frutescens* L. with a spicy taste. An increasing number of studies have shown that capsaicin, in addition to being used in the food sector, also has anti-inflammatory, anticarcinogenic, antiobosogenic and other biological properties. Notably, capsaicin alleviated the severity of CP, including acinar loss, ADM, interstitial fibrosis, and inflammatory cell infiltration, in *LSL-Kras^G12D^/Pdx1-Cre* mice and reduced the number of high-grade PanIN lesions. The main mechanism of the anticancer effects may involve blocking abnormal activation of the phosphate extracellular signal-regulated kinase (ERK) and Hedgehog (Hh)/Gli signalling pathways (Bai et al. 2011).

## Berberine

*Coptis chinensis* Franch. (Huanglian), known for its content of the alkaloid berberine (C_20_H_18_NO_4_^+^), is equally renowned for its ability to purge excessive heat and resolve dampness within the framework of TCM practice. Due to its low bioavailability, it often plays a role in the gastrointestinal system. Berberine has anti-inflammatory effects on inflammatory diseases, including inflammatory bowel disease and hepatitis. Furthermore, it has antagonistic effects on nasopharyngeal carcinoma, stomach cancer, liver cancer and bowel cancer (Zou et al. 2017). Berberine inhibits the development of ADM and PanIN by inhibiting glycolysis and reduces the degree of PF (Liu et al. 2022). Berberine can regulate the cell cycle of PCCs, reduce the viability and proliferation of PCCs, and regulate the production of adenosine triphosphate (ATP) to change the function of mitochondria, thereby inhibiting the occurrence and development of PDAC (Vlavcheski et al. 2022).

## Chinese medicinal formulae composed of botanical drugs for inhibiting PDAC in complex inflammatory microenvironments

Chinese medicinal formulae are composed of multiple botanical drugs in a certain proportion; the combination of drugs is determined according to the principle of the sovereign, minister, assistant and guide. The botanical drugs have the ability to work in concert with other botanical drugs and regulate the body and the disease from a number of perspectives, targets, and relationships. It is the main form by which TCM is used in the clinic. The Chinese medicinal formulae that participate in the inhibition of PDAC in complex inflammatory microenvironments are listed in Table 2.

**Table 2.** The Chinese medicinal formulae composed of botanical drugs for inhibiting PC in complex inflammatory microenvironments.

Formula	Study design	Dosage	Efficacy	Possible mechanism	Refs
Xiao Chaihu Decoction	Male C57BL/6 mice injected cerulein for CP	30 g/kg or 60 g/kg twice a day for 2 weeks	↓Tissue damage; ↓Inflammatory cells infiltration; ↓PF; ↓ECM deposition; ↓Activation of PSCs;	↓α-SMA, Col I, Col III, NLRP3, TNF-α, IL-6, IL-1β; ↑VD_3_, VDR, VD_3_/VDR;	(Zhang G. et al., 2023)
	Syrian golden hamster injected with N-Nitrosobis (2-oxopropyl) amine for pancreatic tumorigenesis model	16 g/kg/day for 8 weeks	↓Pancreatic tumorigenesis; ↓Mitochondria dysfunction;	↓NAD^+^/NADH radio, MDA, 8-OHdG, mtDNA 6mA; ↑ATP, GSH, ALKBH1, NADH dehydrogenase subunits (ND1, ND2, ND4, ND4L,ND5, ND6);	(Cai et al. 2023)
Modified Xiao Chaihu Decoction	Male Wistar rats injected DBTC for CP	10 g/kg/day for 28 days	↓Tissue damage; ↓Inflammatory cell infiltration; ↓PF; ↓Col deposition; ↓ECM deposition;	↓Amylase, lipase, glucose, TGF-β1, TβRII, Smad3, Col I, Col III; ↑BW, the 6-h urine PABA recovery, MMP13;	(Zhang et al. 2013; Zhang et al. 2020)
Da Chaihu Decoction	KunMing mice injected L-arginine for CP	14 g/kg/day for 1 week	↓Tissue damage; ↓Macrophage infiltration; ↓PF; ↓ECM deposition; ↑Pancreas/body weight ratio;	↓Fn, IL-6, MCP-1, MIP-1α	(Duan et al. 2017)
Chaihu Guizhi Ganjiang Decoction	Male Wistar rats injected DBTC for CP	1.44 g/kg BW/dose/day for 3 days	↓Tissue damage; ↓Inflammatory cell infiltration; ↓PF; ↓Col and ECM deposition; ↓Pancreatic autophagy;	↓α-SMA, Col I, Fn, MMP2, TIMP2, LC3B, Atg5, Beclin-1; ↑mTOR, p-mTOR;	(Cui et al. 2021)
	Rat PSCs treated with control serum and Chaihu Guizhi Ganjiang Tang	50% for 24 h	↓Activation and autophagy of PSCs;	↓α-SMA, Col I, Fn, LC3B, Atg5, Beclin-1; ↑p-mTOR, p-JNK;	(Cui et al. 2021)
Dahuang Danshen Decoction	Male SD rats injected DDC for CP	5.48 g/kg/day for 6 weeks	↓Tissue damage; ↓Inflammatory cell infiltration; ↓PF; ↓OS; ↓Endoplasmic reticulum stress; ↓Col deposition;	↓IL-6, TNF-α, α-SMA, Col 1, Col 3, MDA, ROS, Keap-1, GRP78, p-JNK, caspase-12; ↑GSH, SOD, HO-1, Nrf2 (nucleus), GPX1, GST-*π;*	(Liang et al. 2021)

Note: ↓: Decrease or downregulate; ↑: Increase or upregulate.

## Chaihu classical formulae

Xiao Chaihu Decoction recorded in ‘Treatise on Cold Damage Diseases’ is composed of seven botanical drugs: *Bupleurum chinense* DC. (Chaihu), *Scutellaria baicalensis* Georgi (Huangqin), *Panax ginseng* C.A.Mey. (Renshen), *Glycyrrhiza uralensis* Fisch. ex DC. (Gancao), *Pinellia ternate* (Thunb.) Makino (Banxia), *Zingiber officinale* Roscoe (Shengjiang), *Ziziphus jujuba* Mill. (Dazao). The natural compounds that accumulate in Xiao Chaihu Decoction include liquiritin, baicalin, ginsenoside Rb1, ammonium glycyrrhizinate, scutellarin, baicalein, saikosaponin D, saikosaponin A, rutin, and caffecic acid. Xiao Chaihu Decoction can improve pancreatic injury and reduce inflammatory cell infiltration and ECM deposition by inhibiting the activation of the NOD-like receptor family pyrin domain-containing 3 (NLRP3) inflammasome (Zhang G. et al. 2023). It also regulates ATP production by improving ALKBH1/mtDNA 6 mA-mediated mitochondrial dysfunction in animal models of PanIN lesions, thereby inhibiting the occurrence and development of PDAC (Cai et al. 2023). Modified Xiao Chaihu Decoction retains the core botanical drugs of *Bupleurum chinense* DC. (Chaihu), *Pinellia ternate (*Thunb.*) Makino* (Banxia), *Scutellaria baicalensis* Georgi (Huangqin), *Glycyrrhiza uralensis* Fisch. ex DC. (Gancao), and *Prunus persica* (L.) Batsch (Taoren), which can be used to treat abdominal masses, was added. This formula may target the TGF-β1/Sma- and Mad-related protein (Smad) pathway, thereby increasing the weight of rats with CP; reducing serum amylase, lipase, and blood glucose levels; improving inflammatory cell infiltration, acinar degeneration, and PF in pancreatic tissue; significantly increasing the 6-hour urine *para*-aminobenzoic acid (PABA) recovery rate; and improving pancreatic exocrine dysfunction in CP. Moreover, Col degradation is accelerated *via* the upregulation of MMP13 expression (Zhang et al. 2013; Zhang et al. 2020).

The Da Chaihu Decoction is a modified Xiao Chaihu Decoction, with the addition of the core couplet medicinals of Xiao Chengqi Decoction, such as *Rheum officinale* Baill. (Dahuang) and *Citrus × aurantium f. aurantium* (Zhishi). The natural compounds that accumulate in Da Chaihu Decoction include baicalin, kaempferol, sainfuran, quercetin, wogonin, naringenin, luteolin, paeoniflorin, succinic acid, and 6-gingerol, etc. (Xu et al. 2022). Da Chaihu Decoction is recorded in the ‘Treatise on Cold Damage Diseases’ for the treatment of ‘excess of spleen’, which is actually characterized by diarrhoea and vexation and is extremely similar to the clinical manifestations of AP. Studies have shown that Da Chaihu Decoction can significantly improve tissue injury and PF and inhibit macrophage infiltration into the pancreas (Duan et al. 2017).

Chaihu Guizhi Ganjiang Decoction is also derived from the ‘Treatise on Cold Damage Diseases’. This compound is modified by Xiao Chaihu Decoction through the addition of *Trichosanthes kirilowii* Maxim. (Tianhuafen), *Concha Ostreae* (Muli), *Neolitsea cassia* (L.) Kosterm. (Guizhi), and *Zingiber officinale* Roscoe (Ganjiang). The natural compounds that accumulate in Chaihu Guizhi Ganjiang Decoction include liquiritin apioside, liquiritin, isoliquiritin apioside, baicalin, cinnamic acid, wogonoside, glycyrrhizic acid, wogonin, etc. The combination of all the botanical drugs in the formula can assist in the recovery of healthy qi, drive away pathogenic qi, and then protect the spleen and stomach. This formula can effectively ameliorate pathological changes such as inflammatory cell infiltration, structural abnormalities, glandular atrophy, and PF; reduce pancreatic Col deposition and PSCs activation; inhibit pancreatic autophagy in rats with CP; and inhibit PSCs autophagy and activity by activating the c-Jun N-terminal kinase (JNK)/mammalian target of rapamycin (mTOR) signalling pathway (Cui et al. 2021).

## Other formulae

Dahuang Danshen Decoction is composed of *Rheum officinale* Baill. (Dahuang) and *Salvia miltiorrhiza* Bunge (Danshen). *Rheum officinale* Baill. (Dahuang) is a representative medicine for relaxing bowels and decreasing turbidity, and *Salvia miltiorrhiza* Bunge (Danshen) can activate blood and resolve stasis. There are more than 80 natural compounds that accumulate in Dahuang Danshen Decoction, including salvianolic acid A, emodin, baicalein, etc. Dahuang Danshen Decoction alleviates PF, pancreatic tissue oedema and the inflammatory response; regulates the kelch-like ECH-associated protein 1 (Keap-1)/nuclear erythroid-related factor 2 (Nrf2) pathway to alleviate OS damage caused by CP; enhances the body’s antioxidant capacity; and, to some extent, has a dose-dependent effect on improving endoplasmic reticulum stress and reducing PSCs activation and ECM synthesis and deposition (Liang et al. 2021).

## The natural compounds of botanical drugs for inhibiting PDAC in complex inflammatory microenvironments

### Flavonoids

Flavonoids widely exist in natural plants, with C_6_-C_3_-C_6_ as the basic carbon frame that is formed by two benzene rings connected with three carbon atoms, which is the basis for flavonoids to exert antioxidant, antibacterial and other biological activities (Shen et al. 2022). The natural compounds of botanical drugs that participate in the inhibition of pancreatic inflammation and cancer are listed in Table 3.

**Table 3.** Potential effects of the metabolites of the natural compounds of botanical drugs for inhibiting PC in complex inflammatory microenvironments.

Compound category	Compounds	Study design	Dosage of administration	Efficacy	Possible mechanism	Refs
Flavonoids	Quercetin	Female C57BL/6 mice injected Pan 02-luc cells	10 mg/kg every 2 days for 4 injection	↓Tissue damage; ↓PF; ↓Hypoxia; Remodel TME;	↓α-SMA, Wnt 16, Fn, Col I, HIF-1α	(Zhou et al. 2023)
		Pan 02 cell line and NIH 3 T3 cell line cultured with methylcellulose treated with TGF-β	10 μg/mL for 48 h	Remodel TME	↓α-SMA, Wnt 16, Fn, Col I, HIF-1α	(Zhou et al. 2023)
		Human PCC lines PANC-1 and Patu8988	100 μΜ for 24 h	↓Proliferation, migration and invasion of PCCs; ↑Apoptosis and necrotic of PCCs; ↓Colony formation in PCCs; ↓EMT;	↓The ratio of Ki67-positive cells in total cells, Bcl-2, the invasion number of PCCs, migrated rate of PCCs, Acta2, Vim, α-SMA, N-cadherin, type I Col, vimentin, c-Myc, TGF-β1, p-Smad2, p-Smad3, nuclear translocation of Smad2 and Smad3, Smo, Gli2; ↑The proportion of apoptosis and necrotic cells, cleaved caspase-8, cleaved caspase-3, Bax, Cdh1, E-cadherin, Ptch1;	(Guo et al. 2021)
		Male nude mice (BALB/c) injected PANC-1 cells for PDAC in animal xenograft models	75 mg/kg/day for 30 days	↓Growth of PDAC; ↓Metastasis of PDAC;	↓ Proportion of Ki67 positive cells, Bcl-2, type I Col, vimentin, α-SMA, TGF-β1, p-Smad2, p-Smad3, Shh, Gli 2; ↑Cleaved caspase-8, cleaved caspase-3, Bax, E-cadherin, Ptch1;	(Guo et al. 2021)
	Baicalin	Male C57BL/6 mice injected cerulein for CP	100 mg/kg/day, 6 days/week for 2 weeks and 4 weeks	↓Tissue damage; ↓Macrophage infiltration; ↓PF; ↓Col deposition; ↓Activation of PSCs; ↓Tubular complexes;	↓Col 1A1, α-SMA, NF-κB p65, p-NF-κB p65	(Fan et al. 2020)
		Male C57BL/6 mice PSCs treated with TGF-β	50 μg/ml for 12 h and 24 h	↓Activation of PSCs	↓α-SMA, NF-κB p65, TGF-β1, p-TAK, MCP-1	(Fan et al. 2020)
		Mouse BMDMs treated with supernatant from TGF-β1-stimulated PSCs	50 μg/ml for 24 h	↓Macrophage migration		(Fan et al. 2020)
	Puerarin	Male C57BL/6 mice injected caerulein for CP	100 mg/kg/day for 3 weeks	↓Tissue damage; ↓Inflammatory cell infiltration; ↓PF; ↓ECM deposition;	↓TGF-β1, α-SMA, Col 1α 1, Fn, TNF-α, IL-6, p-JNK1/2, p-ERK1/2, p-p38 MAPK; ↑Pancreas weight, GFAP;	(Zeng et al. 2021)
		Human PSCs treated with PAF	100 nM for 24h	↓Proliferation, activation and migration of PSCs	↓PCNA, α-SMA, Fn, p-JNK1/2, p-ERK1/2, p-p38 MAPK; ↑GFAP;	(Zeng et al. 2021)
		Human PCC lines PANC-1 and PATU-8988T	0.5 mM for 24 h	↓Proliferation, migration and invasion of PCCs; ↑Mitochondria-mediated apoptosis of PCCs; ↓Colony formation in PCCs; ↓EMT; ↓mTOR-mediated glucose metabolism;	↓The ratio of Ki67-positive cells in total cells, c-Myc, Bcl-2, α-SMA, p-Akt, p-mTOR, primary respiration, ATP production, maximum respiration, spare respiration, basal glycolysis rate, compensatory glycolysis rate, GLUT1, HIF-1α; ↑Cleaved caspase-8, Bax, E-cadherin;	(Zhu et al. 2021)
		BALB/c nude mice injected PANC-1 cells for PDAC in animal xenograft models	50 mg/kg every three days for 1 month	↓Growth of PDAC	↓Ki67, c-Myc, Bcl-2, α-SMA, HIF-1α, Snail1, Slug, p-Akt, p-mTOR; ↑Cleaved caspase-8, Bax, E-cadherin;	(Zhu et al. 2021)
‑	Luteolin	Female Sprague-Dawley rats injected TNBS for CP	50 mg/kg/day for 28 days	↓Tissue damage; ↓Inflammatory cell infiltration; ↓PF;	↓Hydroxyproline, α-SMA, TGF-β1	(Yu et al. 2018)
		Sprague-Dawley rat PSCs	12.5 mM for 12 hours and 12.5 mM, 50.0 mM for 48 h	↓Proliferation and activation of PSCs; ↑Apoptosis of PSCs;	↓α-SMA, TGF-β1, IL-1β, IL-6, TNF-α, the percentage of cells in S phase; ↑The percentage of cells in G1/G2 phase;	(Yu et al. 2018)
		Human PCC lines SW1990	50 μM and 100 μM for 24 h	↓Proliferation of PCCs; ↑Apoptosis of PCCs;	↓EDU-positive cells ratio, pro caspase-3, PARP; ↑The relative ratio of cells with low MMP, cleaved caspase-3, cleaved PARP;	(Li et al. 2018)
	Rutin	Male albino Wistar rats fed diet containing ethanol and injected cerulein	100 mg/kg for 3 weeks	↓Inflammation; ↓OS;	↓Lipase/amylase ratio, MPO, caspase-1, ASC, IL-1β, IL-18, TNF-α, TBARS, peroxides, OSI; ↑Fecal trypsin, TAC, GSH, GPx, SOD, CAT;	(Aruna et al. 2014)
		human PCC line, PANC-1, SW1990, MIA PaCa-2	5 µg/mL and 10 µg/mL for 24 h and 48 h	↓Proliferation and Migration of PCCs; ↑Apoptosis of PCCs;	↓Cell viability, mobility, MMP9, Bcl2; ↑Bax, caspase-3, cleaved caspase-8, cleaved caspase-9, miR-877-3p, caspase-8;	(Huo et al. 2022)
	total flavonoids extracted from *Psidium guajava L.* leaves	Male C57BL/6 mice injected cerulein for CP	0.372 g/kg and 0.186 g/kg for 2 weeks	↓Tissue damage; ↓Inflammatory cell infiltration; ↓PF; ↓Col deposition;	↓α-SMA, Col I, Col III, NLRP3, caspase-1, IL-1β, IL-18	(Zhang et al. 2021)
	Icariin	Female C57BL/6 mice injected Panc 02 cells into pancreas for mouse orthotopic model of PC	120 mg/kg for 11 days	↓Orthotopic pancreatic tumor progression; ↓Infiltration of PMN-MDSCs into tumors; ↓Number of PMN-MDSCs in the spleen; ↓Infiltration of M2 macrophages in the tumor; ↓Population of M2 macrophages in the spleen;	↓Population of F4/80^+^CD206^+^ cells, the population of Ly6G^+^ Ly6C^−^ cells	(Zheng et al. 2019)
		RAW 264.7 treated with IL-4 for M2 polarization	80 μM for 4 h	↓M2 polarization	↓The number of CD206^+^ cells, Arg 1, MRC1, p-STAT6	(Zheng et al. 2019)
		Panc 02 cells	150 μM for 48 h	↓Proliferation and migration of PCCs; ↑Apoptosis of PCCs;		(Zheng et al. 2019)
Phenolics	Resveratrol	KPC (*LSL-Kras^G12D/+^*, *Trp53^fl/+^*, and *Pdx1-Cre*) mice	50 mg/kg/day for 8 weeks	↓Desmoplastic reaction; ↓Activation of PSCs; ↑Number of apoptotic Panc-1 cells; ↓EMT and invasion of PCCs;	↓α-SMA, HIF-1α, IL-6, SDF-1, VEGF-A, N-cadherin, vimentin, Bcl-2; ↑E-cadherin, the number of apoptotic CM-exposed Panc-1 cells, caspase-3;	(Xiao et al. 2020)
		Human PSCs treated with hypoxic conditions (3% O_2_)	50 μM for 24 h	↓Activation of PSCs	↓α-SMA, HIF-1α, IL-6, SDF-1, VEGF-A	(Xiao et al. 2020)
		Panc-1 and Mia Paca-2 cells treated with hypoxic conditions and CM from PSCs	50 μM for 24 h	↓EMT and invasion of PCCs; ↑Number of apoptotic Panc-1 cells;	↓Vimentin, Bcl-2, N-cadherin, HIF-1α; ↑E-cadherin, cleaved caspase-3, the number of apoptotic CM-exposed Panc-1 cells;	(Xiao et al. 2020)
		Human PSCs treated with H_2_O_2_	50 μM for 24 h	↓Invasion, migration, activation and glycolysis of PSCs	↓α-SMA, ROS, Glut1, HK2, PKM2, LDHA, miR-21, lactate; ↑PTEN protein;	(Yan et al. 2018)
		Panc-1 cocultured with PSCs	50 μM for 24 h	↓Invasion and migration of PCCs		(Yan et al. 2018)
		Male C57/BL6 mice injected cerulein for CP	20 mg/kg/day for 3 weeks	↓Tissue damage; ↓Inflammatory cell infiltration; ↓PF; ↓ECM deposition;	↓p-Akt, TNF-α, α-SMA, Fn1; ↑Pancreas/body weight ratio, Mist1;	(Xia et al. 2018)
		Murine PSC line LTC-14 treated with TGF-β	50 μM for 24 h		↓α-SMA, Fn1, p-Akt, p-p38.;	(Xia et al. 2018)
		Murine AR42J acinar cell line treated with cerulein	50 μM for 24 h	↓AR42J cells apoptotic	↓p-Akt, p-p38	(Xia et al. 2018)
		Rat PSC line LTC-14 treated with TGF-β	50 μM for 24 h	↓Fibrotic and ECM mediators	↓Acta2, Col I-1, Fn 1α, α-SMA, NF-κ B, p-Akt	(Tsang et al. 2015a)
		Rat PSC line LTC-14 treated with TGF-β	20 μM for 24 h	↓Fibrotic mediators; ↓Activation of PSCs;	↓Acta2, Col I-α1, Fn1, NF-κB p65, α-SMA	(Lin et al. 2015)
	Curcumin	Male Wistar rats PSCs treated with PDGF-BB, IL-1β and ethanol	25 μM for 24 h	↓Col deposition; ↓Proliferation and activation of PSCs;	↓The number of PSCs in the S phase, cyclin D1, α-SMA, α_1_(I) procollagen, α1(III) procollagen, type I Col, MCP-1, p-ERK, p-JNK, and p-p38 MAPK, AP-1, MAPK; ↑The percentage of PSCs in the G_0_/G_1_ phase;	(Masamune et al. 2006)
		Human PSCs treated with hypoxic conditions	20 μM for 48 h or 72 h	↓Proliferation, activation and migration of PSCs; ↓Tumor‑stromal crosstalk;	↓α-SMA, IL-6, migrated distance	(Li et al. 2020)
		Human PCC lines BxPC‑3 and Panc‑1 treated with hypoxic conditions and PSC‑CM	20 μM for 48 h	↓Invasive ability of PCCs; ↓EMT of PCCs; ↓Tumor‑stromal crosstalk;	↓p-ERK, p-NF-κB, vimentin, MMP9; ↑E‑cadherin;	(Li et al. 2020)
		Male Wistar rats PSCs treated with PDGF	5 μM for 4 h	↓Proliferation and activation of PSCs	↓p-ERK1/2; ↑HO-1, p-p38 MAPK;	(Schwer et al. 2008)
		Rat PSC line LTC-14 treated with TGF-β	20 μM for 24 h	↓Fibrotic mediators; ↓Activation of PSCs;	↓Acta2, Col I-α1, Fn1, NF-κB p65, α-SMA	(Lin et al. 2015)
	Ellagic acid	Male Wistar rats PSCs treated with PDGF-BB, IL-1β and TNF-α	10 μg/mL and 25 μg/mL for 1 h	↓Proliferation, migration and activation of PSCs; Modulate recruitment and activation of inflammatory cells;	↓p-PDGF β-receptor, p-ERK, p-Akt, p-JNK, p-p38; α-SMA; α1(I)procollagen; α_1_(III)procollagen, MCP-1, activation of AP-1	(Masamune et al. 2005)
		Male WBN/Kob rats received the NIH-07 diet for CP	100 mg/kg BW/day for 10 weeks	↓Tissue damage; ↓Macrophage infiltration; ↓PF; ↓Col deposition;	↓Col, α-SMA, ED-1-positive cells, MPO, TGF-β1; ↑Pancreas/body weight ratio;	(Suzuki et al. 2009)
		Male Wistar rats PSCs stimulated with TGF-β1 or PDGF-BB	10 μg/mL	↓Activation of PSCs; ↑Anti-oxidant action on PSCs;	↓ROS	(Suzuki et al. 2009)
		Balb C Nude mice injected PANC-1 cells	40 mg/kg BW (Monday through Friday for 6 weeks, once daily)	↓PC growth; ↓Proliferation of PCCs in pancreatic tumor tissues; ↑Apoptosis of PCCs in pancreatic tumor tissues; ↓Inflammation in pancreatic tumor tissues; ↓Angiogenesis; ↓EMT;	↓PCNA, Ki67, Bcl-2, Cyclin D1, CDK-2, CDK-6, COX-2, VEGF, VEGFR, HIF-1α, circulating VEGFR2-positive endothelial cells, IL-6, IL-8, Snail, MMP2, MMP9, p-Akt, Gli 1, Gli 2, Notch1, Notch3, Hey1; ↑Activation of caspase-3, cleaved PARP, Bax, E-cadherin, TRAIL-R1/DR4, TRAIL-R2/DR5;	(Zhao et al. 2013)
	Salvianolic acid B	Male Sprague Dawley rats injected DBTC for CP	10 mg/kg for 4 weeks	↓Tissue damage; ↓Inflammation; ↓PF; ↓Pseudotubular complexes; ↓OS; ↓Activation of PSCs;	↓MDA, α-SMA, Col I, TGF-β_1_, p-Smad2/3; ↑SOD, Smad7, the ratio of pancreas wet weight/BW;	(Xu et al. 2016)
		Male Wistar rats PSCs	0.1 mmol/L for 24 h or 48 h	↓Proliferation of PSCs; ↓Represses EMT components;	↓MDA, α-SMA, Col I, Vimentin, TGF-β_1_, p-Smad2/3; ↑SOD, E-cadherin, Smad7;	(Xu et al. 2016)
Terpenoids	Saikosaponin D	Male Wistar rats injected DBTC for CP	2.0 mg/kg BW/day for 4 weeks	↓Tissue damage; ↓Inflammatory cell infiltration; ↓PF; ↓Col deposition; ↓ECM formation and deposition; ↓Activation and autophagy of PSCs;	↓α-SMA, Col I, Fn, Beclin-1, Atg5, LC3B, MMP2, MMP13, TIMP1, TIMP2, TGF-β1, Smad3, p-Smad3; ↑P62, p-PI3K, p-Akt, p-mTOR, the ratio of MMP2/TIMP2, the ratio of MMP13/TIMP1, Smad7;	(Cui et al. 2019)
		Rat PSCs treated with rapamycin	10 μg/mL for 24 h	↓Activation and autophagy of PSCs	↓α-SMA, Col I, Fn, Beclin-1, Atg5, LC3B; ↑p-PI3K, p-Akt, p-mTOR;	(Cui et al. 2019)
		Male Wistar rats injected DBTC for CP	1 mg/kg/day for 4 weeks	↓Tissue damage; ↓Inflammation; ↓Apoptosis of pancreatic cells;	↓IL-6, TNF-α, IL-1β, Bax, cleaved caspase-9, cleaved caspase-3, p-JNK, p-ERK 1/2, p-p38 MAPK	(Li et al. 2021)
		Rat pancreatic acinar AR42J cells treated with cerulein or PSCs-CM or PSCs-CM and cerulein combination	5 μM for 24 h	↓Inflammation; ↓Apoptosis of pancreatic cells;	↓Bax, IL-6, TNF-α, IL-1β, cleaved caspase-9, cleaved caspase-3, p-JNK, p-ERK 1/2, p-p38 MAPK; ↑Bcl-2, pro-caspase-9, pro-cspase-3;	(Li et al. 2021)
		Mouse PCC lines Panc02 and H7 cells	5 μM for 24 h for Panc02; 20 μM for 24 h for H7	↓Proliferation and migration of PCCs; ↑Apoptosis of PCCs;	–	(Xu et al. 2023)
		Primary bone marrow cells stimulated with LPS for M1 polarization or IL-4 for M2 polarization	5 μM for 24 h	↓M2 polarization; ↑M1 polarization;	↓Percentage of F4/80^+^CD206^+^ cells, Arg-1, CD206, TGF-β, IL-10; ↑Percentage of CD86^+^ M1 macrophages;	(Xu et al. 2023)
		Mouse macrophage cell line RAW 264.7 cells stimulated by IL-4 to induce M2 polarization	0.5 μM, 1 μM and 5 μM for 24 h	↓M2 polarization	↓p-STAT6, p-PI3K, p-Akt, p-mTOR	(Xu et al. 2023)
		Female C57BL/6 mice injected Panc02 cells for mouse orthotopic model of PC	1 mg/kg/day for 12 days	↓Orthotopic pancreatic tumor progression; ↓Infiltration of M2 macrophages in the tumor; ↓Population of M2 macrophages in the spleen; ↑Infiltration of CD3^+^/CD8^+^ T cells in tumors; ↓Infiltration of PMN-MDSCs into tumors; ↓Number of PMN-MDSCs in the spleen; Restore NK cell activation;	↓Arg-1, population of F4/80^+^CD206^+^ cells; ↑iNOS, the ratio of CD3^+^ CD8^+^ cells in the tumors;	(Xu et al. 2023)
	Saikosaponin A	Rat PSCs treated with rapamycin and LPS	10 μg/mL for 24 h	↓NLRP3 inflammasome activation; ↓Col deposition; ↓ECM deposition; ↓Viability, proliferation, migration, activation and autophagy of PSCs; ↑ Apoptosis of PSCs;	↓Bcl-2, α-SMA, Col I, Col III, Fn, TIMP1, TIMP2, Atg 5, Beclin-1, LC3B, NLRP3, caspase-1, IL-1β, IL-18, p-AMPK; ↑Bax, MMP13, P62, p-mTOR;	(Cui et al. 2020)
	Triptolide	Hic-5 KO mice (C57BL/6 background) injected caerulein for CP	100 ug/kg/day, six times a week for 4 weeks	↓Tissue damage; ↓Inflammation; ↓PF; ↓ECM deposition;	↓α-SMA, Col 1a1, IL-6, Col 3a1, TIMP1	(Chen et al. 2020)
		Mice PSCs activated by TGF-β	10 nM, 50 nM and 200 nM for 12 and 24 h	↓Activation, proliferation and migration of the PSCs	↓NF-κB/p65, α-SMA, Col 1a1, vimentin	(Chen et al. 2020)
	DA-9601	Male ICR mice injected caerulein for CP	Diets containing 100 mg/kg/day DA-9601 for 10 weeks	↓Tissue damage; ↓PF; ↓OS; ↓ECM deposition; ↓Activation of PSCs;	↓Serum amylase, MDA, iNOS, COX-2, NF-κB; ↑GSH, HSP 70, MT-1;	(Yoo et al. 2005)
		Mice PSCs activated by TGF-β	10 mg/mL	↓Activation of PSCs	↓α-SMA, type I Col	(Yoo et al. 2005)
Alkaloids	Capsaicin	Neonatal Lewis rats injected DBTC for CP	50 mg/kg (pretreatment)	↓Inflammation; ↓PF; ↓Col deposition; ↓Primary sensory denervation;	↓The number and diameter of substance P-immunoreactive nerves	(Ikeura et al. 2007)
		Human PCC lines AsPC-1 and BxPC-3	150 μM for 1 h or 24 h	↓Proliferation of PCCs; ↓Mitochondrial membrane potential; ↑Apoptotic of PCCs;	↓Cells with JC-1, surviving, Bcl-2; ↑Number of apoptotic cells, cells with DCF fluorescence, Bax, cytochrome c, AIF, cleavage caspase-9, cleavage caspase-3, cleavage PARP, ROS, p-JNK;	(Zhang et al. 2008)
		Female athymic nude mice injected AsPC-1 tumor cells	2.5 mg/kg BW five times a week for 39 days and 5 mg/kg three times a week	↓Growth rate of AsPC-1 tumor xenografts; ↑Apoptotic of PCCs;	↓Volume and wet weight of the tumors, count of brown apoptotic bodies; ↑Bax, cytochrome c, AIF, cleavage caspase-3, p-JNK;	(Zhang et al. 2008)
	Matrine	Male SD rats injected TNBS for PF model	100 mg/kg/day for 4 weeks	↓Tissue damage; ↓Inflammatory cell infiltration; ↓PF; ↓Col deposition; ↓Activation and proliferation of PSCs;	↓α-SMA, TGF-β1, Col I, Smad2, TβR1, TβR2	(Liu et al. 2019)
		Human PCC lines 8988T, MIAPACA2	2 mM for 24 h	↓Growth and proliferation of PCCs; Deprive tricarboxylic acid cycle substrates; ↓Mitochondrial metabolism; accumulate autophagic vacuoles; ↓Autophagic degradation;	↓Cell colony, CIT, AKG, FUM, SUC, MAL, ATP, Oxygen consumption rates, Stat3; ↑LC3-II, the number of autophagic vacuoles, p62, LysoTracker signal, cathepsins B, cathepsins D;	(Cho et al. 2018)
Anthraquinones	Rhein	Male C57BL/6 mice injected cerulein for CP	Gavage at 50 mg/kg/day for 6 weeks (prophylactic)	↓Tissue damage; ↓Inflammatory cell infiltration; ↓PF; ↓Col deposition; ↓ECM deposition; ↓Activation of PSCs;	↓TNF-α, IL-1β, Fn1, α-SMA, Col I-α1, TGF-β, Shh, Gli 1, Acta2; ↑Pancreatic weight;	(Tsang et al. 2013)
		Rat PSC line LTC-14 treated with TGF-β or Shh	10 μM and 100 μM for 24 hours for PSCs	↓Fibrogenic mediators; ↓Activation of PSCs;	↓NF-κB, Shh, Gli 1, Fn1, α-SMA, Acta2, Col I-α1	[133] (Tsang et al. 2013)
		Rat PSC line LTC-14 and PANC-1 treated with TGF-β	20 μM for 24 h	↓Fibrosis and EMT mediators	↓Acta2, Col I-α1, Fn1, MMP2, MMP9, CDH2, Shh, α-SMA, Gli 1, p-Akt, NF-κB p65	(Tsang and Bian 2015)
		Rat PSC line LTC-14 treated with TGF-β	20 μM for 24 h	↓Fibrotic mediators; ↓Activation of PSCs;	↓Acta2, Col I-α1, Fn1, NF-κB p65, α-SMA	(Lin et al. 2015)
		Human PCC lines Panc-1 and MIAPaca-2	100 μM for 24 h	↓PCCs growth; ↓Long-term survival ability of PCCs; ↑Apoptotic of PCCs;	↓Colony numbers, CDK4, CDK6, cyclin D1, cyclin E, cells with JC-1, Bcl-2, Bcl-XL, surviving, XIAP, p-Akt, p-PDK1, p-PTEN; ↑The population of G0/G1 cells, p21, p27, the percentages of PCCs in sub-G1 phase, cleaved caspase-9, cleaved caspase-3, cleaved PARP, Bax, Cyt C, ROS;	(Liu et al. 2021)
		Male nude BALB/c mice injected Panc-1 cells	50 mg/kg for 4 weeks	↓Growth of PC; ↑Therapeutic effects of oxaliplatin;	↓The ratio of PCNA-positive cells, p-Akt, Bcl-2, Bcl-XL. ↑The ratio of TUNEL-positive cells, MDA;	(Liu et al. 2021)
	Emodin	Male Sprague-Dawley rats injected TNBS for CP	80 mg/kg BW for 28 days	↓Tissue damage; ↓PF; ↓Col deposition;	↓HA, Ln, Col, TGF-β1	(Wang et al. 2007)
		Rat PSC line LTC-14 treated with TGF-β	4 μM for 24 h	↓Fibrotic mediators; ↓Activation of PSCs;	↓Acta2, Col I-α1, Fn1, NF-κB p65, α-SMA	(Lin et al. 2015)
Glycosides	Amygdalin	Male Wistar rats injected DBTC for CP	10 mg/kg/day from the next day after induction of CP, once a day for the previous 3 days, and then once every 2 days, until 28 days	↓Tissue damage; ↑Pancreatic microcirculation; ↓Inflammation; ↓PF; ↓Activation of PSCs;	↓α-SMA, PDGF-BB, TGFβ-1, ET-1; ↑CGRP, BW, pancreatic blood flow;	(Zhang et al. 2018)
	Eruberin A	Rat PSC line LTC-14 pre-incubated with recombinant TGF-β	20 μg/ml for 24 h	↓Fibrogenic mediator	↓Acta2, TGF-β, Col I-1α, Fn1, α-SMA, NF-κB p65, Shh, Gli1, p-Akt	(Tsang et al. 2015b)
Other compounds	Scoparone	Male Sprague Dawley rats injected DBTC for CP	60 mg/kg for 4 weeks	↓Tissue damage; ↓Inflammation; ↓PF; ↓Pseudotubular complexes; ↓OS; ↓Activation of PSCs;	↓MDA, α-SMA, Col I, TGF-β_1_, p-Smad2/3; ↑SOD, Smad7;	(Xu et al. 2016)
		Male Wistar rats PSCs	0.1 mM, 0.2 mM and 0.4 mM for 24 h or 48 h	↓Represses EMT components	↓MDA, α-SMA, Col I, Vimentin, TGF-β_1_, p-Smad2/3; ↑SOD, E-cadherin, Smad7;	(Xu et al. 2016)
	Withaferin A	C57BL/6 mice injected caerulein for CP	1.25 mg/kg once a week for 4 weeks (preventive) 0.625 mg/ kg twice a week for 4 weeks (curative)	↓Tissue damage; ↓Jnflammatory cell infiltration; ↓PF; ↓Endoplasmic reticulum stress;	↓Amylase, IL-6, monocyte chemotactic protein 1, the percentage of Ki67-positive cells, Nos2, Bax, caspase 3, activation and translocation of NFκB p65, PERK, Atf6, ERN1, Atf4, Xbp1, CHOP, Pycard, NLRP3, IL-18	(Kanak et al. 2017)
	Taurine	Male Sprague-Dawley rats injected TNBS for CP	1000 mg/kg/day for 4 weeks	↓Tissue damage; ↓PF; ↓OS;	↓MDA; ↑GPx, SOD, BW;	(Mas et al. 2006)
	Osthole	Female C57BL/6 mice injected Panc 02 cells into pancreas for mouse orthotopic model of PC	120 mg/kg for 14 days	↓Orthotopic pancreatic tumor progression; ↓Infiltration of M2 macrophages in the tumor and spleen; ↓Infiltration of M2 macrophages in the tumor; ↓Population of M2 macrophages in the spleen;	↓Tumor weight, population of F4/80^+^CD206^+^ cells	(Wang et al. 2018)
		Primary bone marrow cells stimulated with IL-4 for M2 polarization	80 μM for 48 h	↓M2 polarization	↓Percentage of F4/80^+^CD206^+^ cells, MRC1, Ccl22, TGF-β	(Wang et al. 2018)
		RAW 264.7 cells stimulated by IL-4 to induce M2 polarization.	80 μM for 2 h	↓M2 polarization	↓p-STAT6, p-ERK1/2, C/EBP β	(Wang et al. 2018)
		Panc 02 cells	160 mM for 48 h	↓Proliferation, migration of Panc 02 cells; ↑Apoptosis of Panc 02 cells;	↑Proportion of apoptotic cells, wound area	(Wang et al. 2018)

Note: ↓: Decrease or downregulate; ↑: Increase or upregulate.

### Quercetin

In addition to reducing ADM and PanIN lesions, quercetin can also improve the inflammatory microenvironment. It suppresses hypoxia inducible factor-1 (HIF-1α) activation, reduces the expression of Fn and Col, remodels fibrosis, and alleviates the hypoxic tumour microenvironment (Zhou et al. 2023). Additional research on the utilization of quercetin for PC has revealed that quercetin can promote the apoptosis of PCCs, block the proliferation of PCCs, and reduce the activity of the TGF-β1/Smad2/3 and Sonic Hedgehog (Shh) signalling pathways to prevent epithelial-mesenchymal transition (EMT) and the growth of solid tumours from growing and spreading in mice (Guo et al. 2021). Quercetin has been used in clinical trials for Alzheimer’s disease, coronary artery disease and other diseases (Joma et al. 2024). However, the chemical stability of quercetin is affected by factors such as heat and pH. Moreover, the solubility of quercetin in water at 25 °C is only approximately 0.01 mg/mL, and its bioavailability is also low. Long-term administration is necessary to improve its efficacy (Wang et al. 2016).

### Baicalin

Baicalin (C_21_H_18_O_11_) is also one of the most abundant flavonoids in *Scutellaria baicalensis* Georgi (Huangqin) and is formed by the combination of baicalein and a molecule of glucuronic acid (Wang, Wang et al. 2024). It has also been shown to ameliorate local damage to the pancreas in CP by reducing macrophage and inflammatory cell infiltration into the local microenvironment and by reducing the deposition of Col. Downregulation of the TGF-β1/TGF-β receptor 1/TGF-β-activated kinase (TAK1)/NF-κB pathway can suppress the activation of PSCs and significantly ameliorate the severity of pancreatitis and PF (Fan et al. 2020). However, due to its poor water solubility (aqueous solubility of 52 μg/mL), low bioavailability and instability in biological fluids and basic pH, the clinical application of baicalin has been limited (Wang, Wang et al. 2024).

### Puerarin

*Pueraria montana var. lobata* (Willd.) Maesen & S.M. Almeida ex Sanjappa & Predeep (Gegen), containing puerarin (C_21_H_20_O_9_), is recognized for its febrifuge properties and ability to enhance microcirculation. Puerarin has various properties, including anti-inflammatory, pain-relieving and antioxidant properties (Zhou et al. 2014). Puerarin may improve pancreatitis and PF; reduce the ECM; and suppress the activation, proliferation, and migration of PSCs by reducing the levels of phosphorylated mitogen-activated protein kinase (MAPK) family proteins (Zeng et al. 2021). When puerarin is applied for the treatment of PC in a nude mouse xenograft model and *in vitro*, it can inhibit the proliferation and migration of PCCs (Zhu et al. 2021). Additionally, it acts as a treatment for PDAC by causing an imbalance between B-cell lymphoma 2 (Bcl-2) and Bcl-2-associated X protein (Bax), which in turn promotes PCCs apoptosis and inhibits protein kinase B (Akt)/mTOR activity to inhibit glucose uptake and metabolism (Zhu et al. 2021). At present, the main methods of clinical administration are intravenous injection or eye drops, which are often used for cardiovascular diseases and retinal artery and vein occlusion. Due to the low water solubility, poor membrane permeability, and short half-life of puerarin, its bioavailability is poor, which limits its clinical application (Zhang 2019).

### Luteolin

Luteolin (C_15_H_10_O_6_) has various properties, including antioxidant, anti-inflammatory, hepatoprotective, anticancer, and neuroprotective properties. Luteolin has a regulatory function in numerous tissues, including the heart, liver, brain, fat and skeletal muscle. Notably, in the pancreas, luteolin can affect pancreatic β cells and the activity of lipases, thereby affecting insulin secretion and effectively combating glucose and lipid metabolism disorders (Wang et al. 2021). Luteolin significantly reduces the histological characteristics of CP injury, such as glandular atrophy, inflammatory cell infiltration, and PF, reduces the proliferation rate of PSCs, increases the apoptosis rate, and inhibits PSC activation (Yu et al. 2018). Interestingly, luteolin acts as a small molecule inhibitor of Bcl-2, which enables mitochondrial permeability by replacing Bax and triggering apoptosis in SW1990 cancer cells (Li et al. 2018). Currently, the application of luteolin in clinical trials involves diseases such as metabolic syndrome, schizophrenia, arthritis, muscle pain, and COVID-19, etc. However, its water solubility and oral bioavailability are still low, limiting its clinical application (Shi et al. 2024).

### Rutin

Rutin (C_27_H_30_O_16_) is present in a variety of plants, especially *Styphnolobium japonicum* (L.) Schott (sophora rice), *Fagopyrum esculentum* Moench (buckwheat), etc., and has antioxidant and anti-inflammatory biological activities (Muvhulawa et al. 2022). Rutin may be an inhibitor of cysteinyl aspartate specific proteinase-1 (caspase-1) activation by downregulating the expression of apoptosis-related proteins such as apoptosis associated speck-like CARD containing protein (ASC) in the pancreas, thereby regulating NLRP3, reducing the severity of pancreatitis and pancreatic dysfunction, and alleviating the OS response and pancreatic inflammation through its ability to scavenge free radicals (Aruna et al. 2014). In PC, rutin significantly inhibits the proliferation and migration of PCCs and inhibits the transcription of Bcl-2 by upregulating miR-877-3p expression, resulting in the promotion of PCCs apoptosis (Huo et al. 2022). The bioavailability of rutin in humans requires further investigation, which could advance its clinical utility (Muvhulawa et al. 2022).

### Total flavonoids extracted from Psidium guajava L. leaves

*Psidium guajava* L. grows mostly in tropical areas such as Southeast Asia and South America and has a long medicinal history (Naseer et al. 2018). The extract of *Psidium guajava* L. leaves has various biological activities, including antioxidant, hypoglycaemic, and anticancer properties (Kumar et al. 2021). The total flavonoids extracted from *Psidium guajava* L. leaves can effectively alleviate the degree of acinar atrophy and inflammatory cell infiltration, reduce Col deposition, which is mainly composed of type I Col, reduce the population of activated PSCs, and prevent the activation of NLRP3 inflammasomes to alleviate the degree of PF (Zhang et al. 2021).

### Icariin

Icariin (C_33_H_40_O_15_), a major active component of *Epimedium sagittatum* (Siebold & Zucc.) Maxim. (Yinyanghuo), has a wide range of pharmacological effects, including enhancing sexual desire, preventing osteoporosis, regulating immune function, etc. (Bi et al. 2022). Studies have demonstrated that icariin blocks the progression of PC by inducing apoptosis and inhibiting the proliferation and migration of PCCs. Moreover, icariin also regulates the immune microenvironment by downregulating the signal transducer and activator of the transcription 6 (STAT6) signalling pathway and reducing inflammatory cell infiltration in tumour tissue even if macrophages transform into M2 cells (Zheng et al. 2019). However, due to the short half-life and poor bioavailability of icariin, there is an urgent need to develop more effective delivery methods and drug delivery systems (Bi et al. 2022).

### Phenolics

Phenolics commonly present in plants are an important part of the human diet. They can be described as a class of compounds with a phenol structure containing one or more hydroxyl groups, which has strong antioxidant properties (H.H. Al Mamari 2022; Shahidi and Ambigaipalan 2015).

### Resveratrol

Resveratrol improves the integrity of acinar cells and immune cell infiltration, inhibits the activation of PSCs under hypoxic conditions, and alleviates the proconnective tissue proliferation response by reducing the production of fibrosis mediators and ECM proteins. Inhibiting ROS/miR-21-mediated glycolysis and activation in PSCs may hinder the invasion and migration of PCCs (Lin et al. 2015; Xiao et al. 2020; Yan et al. 2018; Xia et al. 2018; Tsang et al. 2015a). Numerous studies have shown that resveratrol can induce cell cycle arrest, inhibit the proliferation of PCCs by inhibiting the Hh signalling pathway and phosphoinositide 3-kinase (PI3K)/Akt signalling pathway, and control Bcl-2 in multiple pathways to cause PCCs to undergo apoptosis. Furthermore, it can suppress the invasion and metastasis of PCCs, increase the sensitivity of tumours to radiotherapy and chemotherapy, and inhibit the self-renewal ability of PC stem cells (Xu et al. 2015; Shankar et al. 2011). Resveratrol has been used in clinical trials for the treatment of type 2 diabetes mellitus insulin resistance, prostate cancer, colorectal cancer and other diseases (Joma et al. 2024). However, the bottleneck of resveratrol application lies in its low solubility and bioavailability (Berman et al. 2017; Salehi et al. 2018).

### Curcumin

*Curcuma longa* L. (Jianghuang), a botanical drug that has been used in both medicine and food for thousands of years, is the main source of curcumin (C_21_H_20_O_6_) and is valuable for its ability to promote blood circulation and resolve stasis. Curcumin is often used in beverages and food in many forms. In terms of its medicinal value, curcumin has good safety and has antioxidant, anti-inflammatory and other effects. It may effectively prevent PSCs from multiplying, increase the percentage of G0/G1 phase cells, decrease the number of S phase cells by downregulating cyclin D1 expression, and improve the state of Col deposition by decreasing the expression of α-SMA and Col (Masamune et al. 2006). Curcumin inhibits the activation and migration ability of PSCs in hypoxic environments and reduces the proliferation by upregulating haem oxygenase-1 (HO-1) expression (Li et al. 2020; Schwer et al. 2008). It may also decrease the expression of the PSCs fibrotic mediators encoding α-SMA (Acta2), Col I-1α, and Fn 1 and inhibit NF-κB signal transduction (Lin et al. 2015). In addition, curcumin may block the production of monocyte chemoattractant protein-1 (MCP-1) by inhibiting MAPK, thereby reducing the ability to recruit and activate inflammatory cells (Masamune et al. 2006). In this complex microenvironment, curcumin can also be used to prevent the activation of the IL-6/ERK/NF-κB axis to effectively reduce the invasive ability of PCCs in PSCs-conditioned medium (CM) under hypoxia, effectively increase E-cadherin levels, reduce the expression of vimentin and MMP9, and inhibit the EMT of PCCs (Li et al. 2020). Curcumin has been used in women with polycystic ovary syndrome (PCOS) to improve hyperglycaemia and dyslipidaemia associated with PCOS (Wang, Zhang, et al. 2024). However, due to the low solubility and poor stability of curcumin, its bioavailability is poor. Therefore, it is necessary to solve this dilemma by synthesizing curcumin analogues and other methods (Nagaraju et al. 2019).

### Ellagic acid

Ellagic acid (C_14_H_6_O_8_), which is widely found in fruits and vegetables, is thought to have a more powerful antioxidant effect than both melatonin and vitamin E (Zhu et al. 2022). Ellagic acid can significantly alleviate lesions such as PF in CP; inhibit the activation of PSCs by suppressing the MAPK pathway and ROS production; reduce the proliferation, migration, and transformation of PSCs into a myofibroblast-like phenotype; reduce the accumulation of Col; and reduce the infiltration of white blood cells (Masamune et al. 2005; Suzuki et al. 2009). Additionally, ellagic acid may limit PC growth and metastasis through the inhibition of the Akt, Notch and Shh pathways, reducing angiogenesis in the tumour microenvironment (Zhao et al. 2013). The bioavailability of tannic acid is extremely low, and it is often metabolized into a more bioavailable urolithin under the action of the gut microbiota. Therefore, it is necessary to conduct in-depth basic and clinical research on the relationship between tannic acid and the gut microbiota, as well as the biological function of urolithin (Zhang M. et al. 2023).

### Salvianolic acid B

*Salvia miltiorrhiza* Bunge (Danshen), which contains the phenolic compound salvianolic acid B (C_36_H_30_O_16_), is also highly valued for its ability to promote blood circulation and resolve stasis. Salvianolic acid B has been used to treat liver disease, kidney disease, bone disease, diabetes and other diseases because of its multiple biological activities (He et al. 2023). Salvianolic acid B inhibits the activation of TGF-β/Smad signal transduction, suppresses PSCs activation and proliferation, reduces EMT, decreases OS levels, prevents pancreatic damage and reduces the severity of PF (Xu et al. 2016). At present, clinical trials of salvianolic acid B have been conducted for the treatment of coronary heart disease and angina pectoris. However, as a hydrophilic compound, its stability in water is poor, and it is difficult to achieve long-term stable release and ideal therapeutic effects (He et al. 2023).

### Terpenoids

Terpenoids are based on isoprene, which is composed of five carbon atoms as a skeleton, and most of them have polycyclic structures with various biological activities, such as anticancer, antibacterial and antioxidant activities (Masyita et al. 2022).

### Saikosaponin D

*Bupleurum chinense* DC. (Chaihu), a cornerstone in TCM for harmonizing the Shaoyang meridian and reducing fever. The classic Chaihu formula plays an essential role in suppressing pancreatitis-induced PDAC, and for treating illnesses such as influenza, malaria, and hepatitis, *Bupleurum chinense* DC. (Chaihu) is widely used. Triterpenoid saponins are the main effective natural compounds of *Bupleurum chinense* DC. (Chaihu). Currently, *Bupleurum chinense* DC. (Chaihu) is currently the source of more than 30 saponins; among them, saikosaponin D (C_42_H_68_O_13_) has anti-inflammatory, antitumour, antialgenic and other biological properties (Yuan et al. 2017). Saikosaponin D may reduce pancreatic injury and inflammation through the inhibition of MAPK pathway phosphorylation and the reduction of pancreatic cell apoptosis. It inhibits the activation of PSCs and PF through activation of the PI3K/Akt/mTOR pathway and inhibition of the TGF-β1/Smad pathway and promotes ECM breakdown by increasing the MMP/TIMP ratio; there may be crosstalk between the two signalling pathways, jointly alleviating the PF of CP (Cui et al. 2019; Li et al. 2021). In addition, saikosaponin D plays a critical role in the regulation of immune cells in PDAC by suppressing p-STAT6 and the PI3K/Akt/mTOR signalling pathway to reduce macrophage polarization into the M2 phenotype, thereby regulating the immunosuppressive microenvironment (Xu et al. 2023). However, saikospaponin D exhibited certain toxic side effects, and after one week of administration at a concentration of 300 mg/kg, the liver tissue of the mice was damaged (Zhang et al. 2016).

### Saikosaponin A

Saikosaponin A (C_42_H_68_O_13_) is mainly extracted from *Bupleurum chinense* DC. (Chaihu) and has a similar structure to saikosaponin D. It has anti-inflammatory, antitumour, neuroregulatory and immunomodulatory properties (Yuan et al. 2017; Yang et al. 2017). Saikosaponin A decreases the activity, proliferation, migration and activation of PSCs and promotes their apoptosis. It inhibits the NLRP3 inflammasome by modulating the 5′-adenosine monophosphate-activated protein kinase (AMPK)/mTOR pathway to inhibit PSCs autophagy, thus exerting therapeutic effects on PF in CP (Cui et al. 2020).

### Triptolide

Triptolide (C_20_H_24_O_6_) is an important diterpenoid active compound in *Tripterygium wilfordii* Hook. f. (Leigongteng), which possesses numerous significant biological properties, including immunosuppressive, anti-inflammatory, and anticancer effects. Triptolide weakens the activation and metastasis of PSCs and reduces the degree of PF by inhibiting NF-κB/p65 expression (Chen et al. 2020) and inhibits EMT by regulating the apoptosis, autophagy and gene expression of tumor cells (Noel et al. 2019). Unfortunately, it has toxic side effects on the liver, kidneys, heart, and reproductive system, and the yield of plant extracts is extremely low. Chemical synthesis is also relatively difficult, limiting its clinical application (Gao et al. 2021). However, combined with its excellent anti-inflammatory effect, triptolide may have a more profound impact on the immune microenvironment of PDAC, which is worthy of further study (Noel et al. 2019).

### DA-9601

DA-9601 is a standardized extract from *Artemisia asiatica* Nakai that has gastric protective and anti-ulcer effects (Ahuja et al. 2018). Research has shown that DA-9601 can alleviate the OS response, reduce ECM deposition, decrease PSCs activation, and improve PF and atrophy (Yoo et al. 2005).

### Alkaloids

Alkaloids contain at least one nitrogen atom as their general chemical characteristics, often alkalinity, and the nitrogen atoms are mostly contained in the ring (Dostál 2000).

### Capsaicin

In addition to reducing ADM and high-grade PanIN lesions, capsaicin can also improve the inflammatory microenvironment. Neonatal rat pretreatment with capsaicin can reduce the infiltration of inflammatory cells and fibrosis in the pancreas by reducing the activation of primary sensory neurons and the release of proinflammatory neuropeptides induced by pancreatic tissue damage (Ikeura et al. 2007). Capsaicin can induce the generation of ROS and activation of JNK, leading to sustained disruption of the mitochondrial membrane potential, thereby increasing the apoptosis of PCCs and inhibiting tumour growth (Zhang et al. 2008). The clinical benefits of capsaicin include improving blood glucose and lipid metabolism disorders, improving obesity, and showing great potential in clinical pain relief. However, its limited bioavailability and short plasma half-life limit its use as a ‘stimulating’ substance. Capsaicin can cause certain adverse reactions and adverse heat sensation, which limits its clinical application (Iftinca et al. 2021; Yasin et al. 2023).

### Matrine

In TCM*, Sophora flavescens* Aiton (Kushen) has the functions of clearing heat, drying dampness and killing worms. As its main chemical component, matrine (C_15_H_24_N_2_O) has shown great clinical application prospects for PC, lung cancer, liver cancer and other cancers and has good anti-inflammatory, antioxidant, antiviral and other biological activities (You et al. 2020). By inhibiting the TGF-β/Smad pathway, matrine may reduce PSCs proliferation and activation, thereby inhibiting fibrosis, reducing mitochondrial swelling and inflammatory cell infiltration, and restoring pancreatic injury to a greater extent (Liu et al. 2019). By interfering with the function of the lysosomal protease, matrine inhibits autophagy, thereby inhibiting the energy metabolism of mitochondria and reducing the growth of PDAC (Cho et al. 2018). However, matrine has limited bioavailability and serious side effects, including hepatotoxicity, neurotoxicity, and reproductive and developmental toxicity; thus, caution is needed in its clinical application (You et al. 2020).

### Anthraquinones

Anthraquinones use 9,10‐anthraquinone with three rings, A, B, and C, due to the addition of a phenol hydroxyl group and other auxochromic groups, which are often used in dyeing. Modern pharmacological studies have shown that it has many biological activities, such as anti-inflammatory, anticancer and antidiabetes effects (Wang et al. 2023).

### Rhein

Rhein (C_15_H_8_O_6_) is derived mainly from traditional Chinese herbs such as *Rheum officinale* Baill. (Dahuang) and *Aloe vera* (L.) Burm.f. (Luhui), both of which are characterized by a cold nature and bitter taste, and they have good heat-clearing effects. Rhein possesses a multitude of pharmacological activities, including anti-inflammatory, antitumour, antioxidant and antifibrotic effects, through its actions on several pathways, such as the MAPK, Akt and NF-κB pathways (Cheng et al. 2021). Rhein inhibits Shh/Gli 1 signal transduction, which may reduce pancreatic gland atrophy, interstitial space enlargement, inflammatory cell infiltration, PSCs activation, and ECM synthesis in the pathogenesis of PF (Lin et al. 2015; Tsang et al. 2013; Tsang and Bian 2015). Rhein can induce the production of ROS and inactivate Akt, thereby inhibiting the PI3K/Akt signalling pathway and activating caspase cascades and the mitochondrial apoptosis pathway, thereby increasing the apoptosis of PCCs. Moreover, rhein also enhances the antitumour effect of oxaliplatin and simultaneously reduces the expression of oncogenic mediators such as MMP2 and MMP9 (Liu et al. 2021). However, owing to the poor solubility and bioavailability of rhein, its application is limited, making the development of derivatives and delivery systems for emodin more promising (Cheng et al. 2021).

### Emodin

*Rheum officinale* Baill. (Dahuang) is celebrated for its ability to clear heat and purge the bowels. *Polygonum cuspidatum* Sieb. et Zucc. (Huzhang) is highly esteemed for its remarkable ability to dispel dampness. Emodin (C_15_H_10_O_5_), the quintessential compound found within both botanical drugs, embodies their shared essence and therapeutic prowess and has a multitude of biological effects, including anticancer, anti-inflammatory, antibacterial and immunosuppressive properties. It is often used in the research of PC, liver cancer and breast cancer (Dong et al. 2016). Emodin effectively reduces the expression of the PSCs fibrotic mediators Acta2, Col I-1α, and Fn 1 in the pancreas and inhibits NF-κB signal transduction to improve PF and pathological damage (Lin et al. 2015; Wang et al. 2007). Emodin has liver, kidney, and male reproductive system toxicity. Moreover, it is quickly eliminated and poorly absorbed in the intestine and has low bioavailability in the body. In future research, attention should be given to the inhibition of glucuronidation metabolism and the toxic side effects of emodin (Dong et al. 2016).

### Glycosides

The hemiacetal hydroxyl group is one of the main symbols of glycosides and has many application prospects, such as anticancer, cholesterol-lowering, and antioxidant effects (Shen et al. 2023).

### Amygdalin

The dry and mature seeds of *Prunus armeniaca* L. (Kuxingren) are often used to treat lung and intestinal diseases. However, when the dosage and administration are not appropriate, a series of adverse reactions, such as nausea, vomiting, diarrhoea, respiratory failure, and arrhythmia, occur. Amygdalin (C_20_H_27_NO_11_) is a rich glycoside compound in the dry and mature seeds of *Prunus armeniaca* L. (Kuxingren), which is not only the main toxic component but also its main pharmacological active ingredient (Tang et al. 2024). Anti-inflammatory, antioxidant, antitumour and pain-relieving properties are among the main pharmacological effects of amygdalin. Amygdalin increases body weight in dibutyltin dichloride (DBTC)-induced CP rats, reduces acinar cell destruction and the degree of PF, increases pancreatic blood flow, and inhibits PSCs activation (Zhang et al. 2018). It is often used in clinical practice for respiratory diseases such as cough, but amygdalin is metabolized in the body and releases hydrogen cyanide, which can be toxic to the body. Improper use may be harmful to the disease itself and not beneficial (Tang et al. 2024).

### Eruberin A

Eruberin A (C_24_H_28_O_9_) is a glucoside that was discovered from the botanical drug *Thelypteris penangiana* (Hook.) C.F.Reed. In actuality, few people are aware of fern as a traditional Tujia medicinal remedy (Zhou et al. 2019). Eruberin A inhibits the production of fibrogenic mediators and the ECM in PSCs and PCCs by inhibiting the PI3K/Akt/NF-κB pathway (Tsang et al. 2015b).

## Other compounds

### Scoparone

Scoparone (C_11_H_10_O_4_), the principal bioactive constituent of *Artemisia capillaris* Thunb. (Yinchen) (Li et al. 2010), bestows upon it the ability to clear and dissipate damp heat and displays a range of biological characteristics, including antioxidant, anti-inflammatory, antifibrotic and hypolipidaemic effects (Hui et al. 2020). Research has indicated that the potential efficacy of scoparone is approximately equivalent to that of salvianolic acid B, which can prevent pancreatic injury and reduce the degree of PF by inhibiting the activation of TGF-β/Smad signal transduction, inhibiting EMT, and reducing the level of OS (Xu et al. 2016). Scoparone lacks selectivity in target selection, and its combination with other drugs may be more effective than a single application, which may require multiple verifications *in vivo*, *in vitro*, and even in clinical trials (Hui et al. 2020).

### Withaferin A

*Withania somnifera* (L.) Dunal mainly grows in India, the Middle East, and Africa and is often used in the Ayurvedic medical system as an astringent and anticarbuncle (Paul et al. 2021). Withaferin A (C_28_H_38_O_6_), a steroid, inhibits PCCs invasion and metastasis and controls apoptosis, autophagy, and the cell cycle. These actions collectively have anticancer properties. It also has various biological activities, namely, anti-inflammatory, anticoagulant, neuroprotective, and hypoglycaemic effects (Xing et al. 2023). Research has shown that withaferin A can effectively slow the progression of CP and alleviate pancreatic damage, regardless of prophylactic administration or treatment, by reducing the expression of NF-κB and proinflammatory cytokines and reducing the expression of endoplasmic reticulum stress proteins (Kanak et al. 2017). The yield of withaferin A is low, its bioavailability is poor, and it has certain toxic side effects. It may be necessary to seek derivatives of withaferin A and develop drug delivery systems to address these limitations (Xing et al. 2023).

### Taurine

Taurine (C_2_H_7_NO_3_S) is an amino acid isolated from *Calculus bovis* (Niuhuang) and is also found in marine plants such as *Porphyra dentata* (Aung et al. 2022). Taurine can resist OS and maintain mitochondrial function (Yu et al. 2020; Jong et al. 2021). Studies have shown that taurine deficiency may be closely related to increased DNA and mitochondrial function damage; thus, taurine deficiency may be the driving factor of ageing (Singh et al. 2023). Taurine reduces the degree of PF in the CP by improving the OS state (Mas et al. 2006). Taurine is widely used in clinical practice because of its low toxicity and easy absorption, which provides broad prospects for future research and application (Calabrese et al. 2024).

### Osthole

As a coumarin compound, osthole (C_15_H_16_O_3_) can be isolated from *Cnidium monnieri* (L.) Cuss. (Shechuangzi), which has antioxidant, anti-inflammatory, anticancer, antihyperglycaemic and neuroprotective pharmacological properties (Zafar et al. 2021). *Cnidium monnieri* (L.) Cuss. (Shechuangzi) has excellent effects on drying dampness. Osthole has an antitumour effect on PDAC by increasing apoptosis and suppressing the proliferation and migration of Panc02 cells. It may play an immunomodulatory role in M2 macrophages by downregulating the STAT6 and CCAAT/enhancer binding protein β (C/EBP β) signalling pathways and decreasing the invasion of tumour tissue by M2 macrophages (Wang et al. 2018). Osthole has the characteristics of low toxicity and diverse targets, but its pharmacokinetic characteristics need further elaboration (Zafar et al. 2021).

## Conclusions and outlook

In this review, our focus is on the possible mechanisms of pancreatitis-induced PDAC and the possible blocking mechanisms of botanical drugs and their natural compounds in this process. In short, at the organ, cell, or molecular level, there may be changes that support the development of PDAC in a sustained inflammatory environment, and both Chinese medicinal formulae and the natural compounds of botanical drugs may have a blocking effect on pancreatitis-induced PDAC. Chinese medicinal formulae are characterized by multiple levels, channels and targets for preventing the occurrence of pancreatitis-induced PDAC. In addition to the natural compounds mentioned above, which can be explained based on TCM theory, the potential of other natural compounds derived from botanical drugs merits consideration. Compounds such as quercetin, resveratrol, rutin, and ellagic acid, which are ubiquitous in nature, possess robust anti-inflammatory or antioxidant capabilities. These attributes render them promising therapeutic agents for the prevention of pancreatitis-induced PDAC, underscoring the importance of a comprehensive evaluation of the pharmacological repertoire of botanical drugs in TCM. The application of Chinese medicinal formulae and the natural compounds of botanical drugs fully demonstrates the potential advantages and good development prospects of botanical drugs, which have been proven to improve the inflammatory microenvironment by improving tissue damage, alleviating the inflammatory response, reducing the level of PF, reducing Col and ECM deposition, improving OS, and restoring glycolysis to improve energy metabolism. More importantly, Chinese medicinal formulae and natural botanical drugs can affect the differentiation of normal cells and reduce the occurrence of ADM and/or PanIN lesions, which is highly important for preventing pancreatitis-induced PDAC.

Due to the complexity of Chinese medicinal formulae and the diversity of chemical structures and pharmacological effects of the natural compounds used in botanical drugs, fully elucidating the specific mechanism by which they block pancreatitis-induced PDAC is difficult. This suggests that this subject needs further study and exploration. In this regard, we propose the following suggestions: (1) In-depth exploration of Chinese medicinal formulae combined with TCM disease differentiation and syndrome differentiation systems and the use of modern medicine to clarify chemical components to find potential drugs that can block pancreatitis-induced PDAC; (2) Further exploration of the natural compounds of botanical drugs with limited application, such as poor solubility, low bioavailability and high toxicity, in terms of dosage and chemical structure modification to provide a basis for more extensive application; and (3) The combined application of various compounds may increase the therapeutic effect, which requires more experimental verification; (4) In experimental research, *in vivo* animal models and *in vitro* cell models should be combined as much as possible, and a variety of modelling methods should be used to jointly promote research on pathogenesis and TCM prevention; (5) Where conditions permit, clinical studies should also be undertaken to investigate the efficacy and safety of botanical drug therapies. We hope that this comprehensive review, which elucidates the potential of botanical drugs and their natural compounds to suppress the precursors of PDAC in both *in vitro* and *in vivo* settings, will serve as a catalyst for advancing the clinical utility of botanical drugs in the management of pancreatitis-induced PDAC.

## Data Availability

All data are fully available without restriction.
